# Molecular Breeding of a Novel PTGMS Line of WDR for Broad-Spectrum Resistance to Blast Using *Pi9*, *Pi5*, and *Pi54* Genes

**DOI:** 10.1186/s12284-021-00537-1

**Published:** 2021-11-25

**Authors:** Yi Liu, Fenyun Zhang, Xingxing Luo, Deyan Kong, Anning Zhang, Feiming Wang, Zhongquan Pan, Jiahong Wang, Junguo Bi, Lijun Luo, Guolan Liu, Xinqiao Yu

**Affiliations:** Shanghai Agrobiological Gene Center, Shanghai, 201106 People’s Republic of China

**Keywords:** Photoperiod and thermo-sensitive genic male sterile line (PTGMS), Blast resistance, Marker-assisted selection, Water-saving and drought-resistance rice (WDR)

## Abstract

**Background:**

The two-line method based on the photoperiod and thermo-sensitive genic male sterile (PTGMS) lines is more cost-effective, simple, and efficient than the three-line system based on cytoplasmic male-sterility. Blast and drought are the most prevalent biotic and abiotic stress factors hampering rice production. Molecular techniques demonstrate higher efficacy in the pyramiding of disease resistance genes, providing green performance under the background of water-saving and drought-resistance rice.

**Results:**

This study employed molecular marker-assisted selection, conventional hybridization, and high-intensity stress screening to integrate three broad-spectrum blast resistance genes *Pi9*, *Pi5*, and *Pi54* into Huhan 1S. Subsequently, a novel water-saving and drought-resistance rice (WDR) PTGMS line Huhan 74S was developed. The drought resistance of the new PTGMS line Huhan 74S was comparable to that of Huhan 1S. Pathogenicity assays involving the inoculation of 14 blast prevalent isolates in the glasshouse showed that the blast resistance frequency of Huhan 74S was 85.7%. Further evaluation under natural blast epidemic field conditions showed that Huhan 74S and its hybrids were resistant to leaf and neck blast. The critical temperature point of fertility-sterility alteration of Huhan 74S was 23 °C daily mean temperature. The complete male sterility under natural growth conditions in 2017 at Shanghai lasted for 67 days. Also, both the agronomic and grain quality traits met the requirement for two-line hybrid rice production.

**Conclusion:**

These results indicate that the newly bred PTGMS line Huhan 74S can be used to breed high-yielding, good-quality, disease-resistant two-line hybrid water-saving and drought-resistance rice (WDR), hence promoting sustainable rice production in China.

**Supplementary Information:**

The online version contains supplementary material available at 10.1186/s12284-021-00537-1.

## Introduction

Rice blast caused by *Magnaporthe oryzae* is a global fungal disease that severely affects rice production and grain quality. Rice blast alone can cause annual yield losses of between 10 and 30% of the total rice harvest. (Skamnioti and Gurr 2009), thereby significantly jeopardizing food security. Breeding and planting various disease-resistant rice are the most economical and efficient measures to control blast. Because of the numerous physiological species and rapid mutation of the blast fungus, most rice varieties have lost their resistance to blast. It is extremely difficult to select broad-spectrum disease-resistant varieties via conventional breeding methods. There is a greater risk of using single-gene to prevent and control rice diseases because the loss of single-gene resistance could lead to the outbreak of larger diseases. Therefore, pyramiding of multiple disease resistance genes is the most feasible and effective approach for solving the current hazard of rice blast (Orasen et al. 2020). So far, over 130 major genes of blast resistance have been identified, thirty of which have been molecularly cloned (Yin et al. 2021). Also, more than 350 quantitative trait loci associated with rice blast resistance have been identified (Li et al. 2020), providing a possibility of using molecular markers to detect these genes. Molecular marker-assisted selection (MAS) screens target traits in advance to enhance breeding efficiency (Tanksley et al. 1989). The *Pi9* gene confers broad-spectrum blast disease resistance in rice. It is located in the *Piz* locus together with similar genes such as *Pi2*, *Pi50*, *Pigm*, *Piz*, and *Piz-t*. Lines carrying the *Pi9* gene showed resistance to 43 rice blast isolates detected from 13 countries (Liu et al. 2002). Also, *Pi5,* located on chromosome 9 conferred resistance to five rice blast isolates, including PO6-6 under artificial inoculation conditions in the greenhouse, in rice varieties with broad spectrum resistance to blast isolates from rice regions in Northeast China (Lee et al. 2009). *Pi54*, a major dominant resistance gene, conferred broad-spectrum resistance against geographically diverse isolates of *M. oryzae* (Sharma et al. 2010; Rai et al. 2011). Many successful marker-assisted introgressions using the *Pi54* gene have been made in diverse backgrounds of rice varieties and hybrids (Ramalingam et al. 2020; Ponnuswamy et al. 2020). These blast resistance genes are significantly valuable in rice breeding for disease resistance.

Photoperiod and thermo-sensitive genic male sterile line (PTGMS) have greatly contributed to increasing rice yields in China (Yuan. 2017). Nonetheless, most PTGMS lines have poor resistance to blast (Dong et al. 2010). Related studies have shown that the disease resistance of hybrid rice is significantly and positively correlated with that of sterile lines (Jiang et al. 2012). As one of the adverse effects of climate change, drought has emerged as an important limiting factor for agricultural production in recent years. Therefore, cultivating water-saving and drought-resistance rice (WDR) is one of the critical strategies to addressing water shortage issues and increasing the yield of low- and medium-yield fields in China (Luo 2010). The two-line sterile line bred in production lacks the characteristics of water-saving and drought-resistance. The breeding history and genomic studies indicate that lowland paddy rice and upland rice hybridization breeding with suitable selection in different environments effectively improves complex traits such as yield potential and drought resistance. Meanwhile, molecular technology has demonstrated higher efficiency in value-added breeding, such as transfer and pyramiding of disease and insect resistance genes, assisting WDR to obtain other green characteristics (Luo et al. 2019). Huhan 1S is a WDR PTGMS line with strong drought resistance but is susceptible to blast. We adopted MAS and conventional hybridization combined with high-intensity stress selection in the target to integrate three broad-spectrum blast resistance genes *Pi9*, *Pi5*, and *Pi54* into Huhan 1S. Subsequently, a novel PTGMS line Huhan 74S with broad-spectrum blast resistance, strong drought resistance, good rice grain quality, and satisfactory combining ability was selected. The line passed the technical appraisal test by the Shanghai Seed Management Station in August 2018, thereby providing an effective parental basis for further grouping novel combinations of disease-resistant two-line hybrid WDR.

## Results

### Breeding Process

**Fig. 1:**
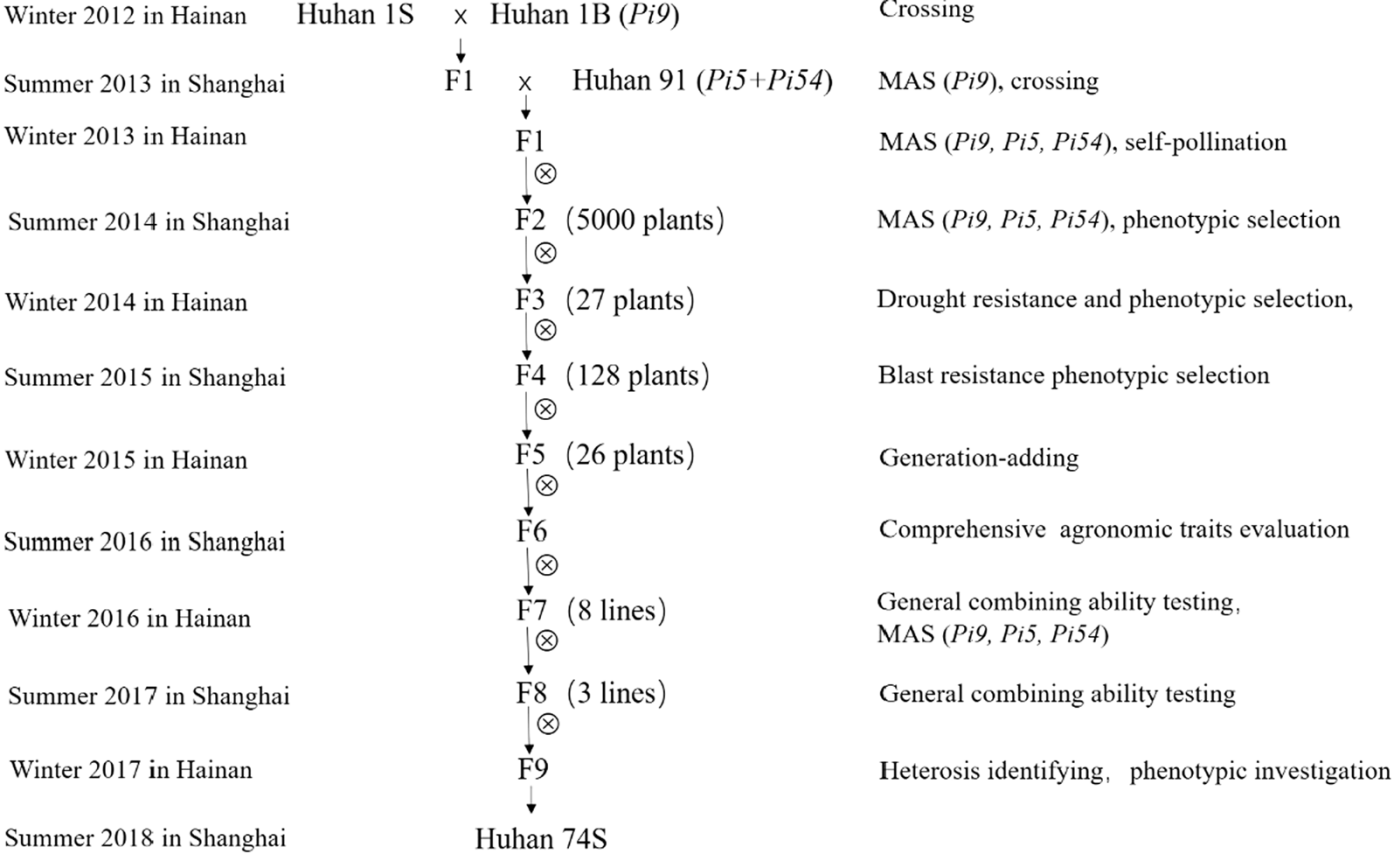
Breeding process of Huhan 74S

**Fig. 2:**
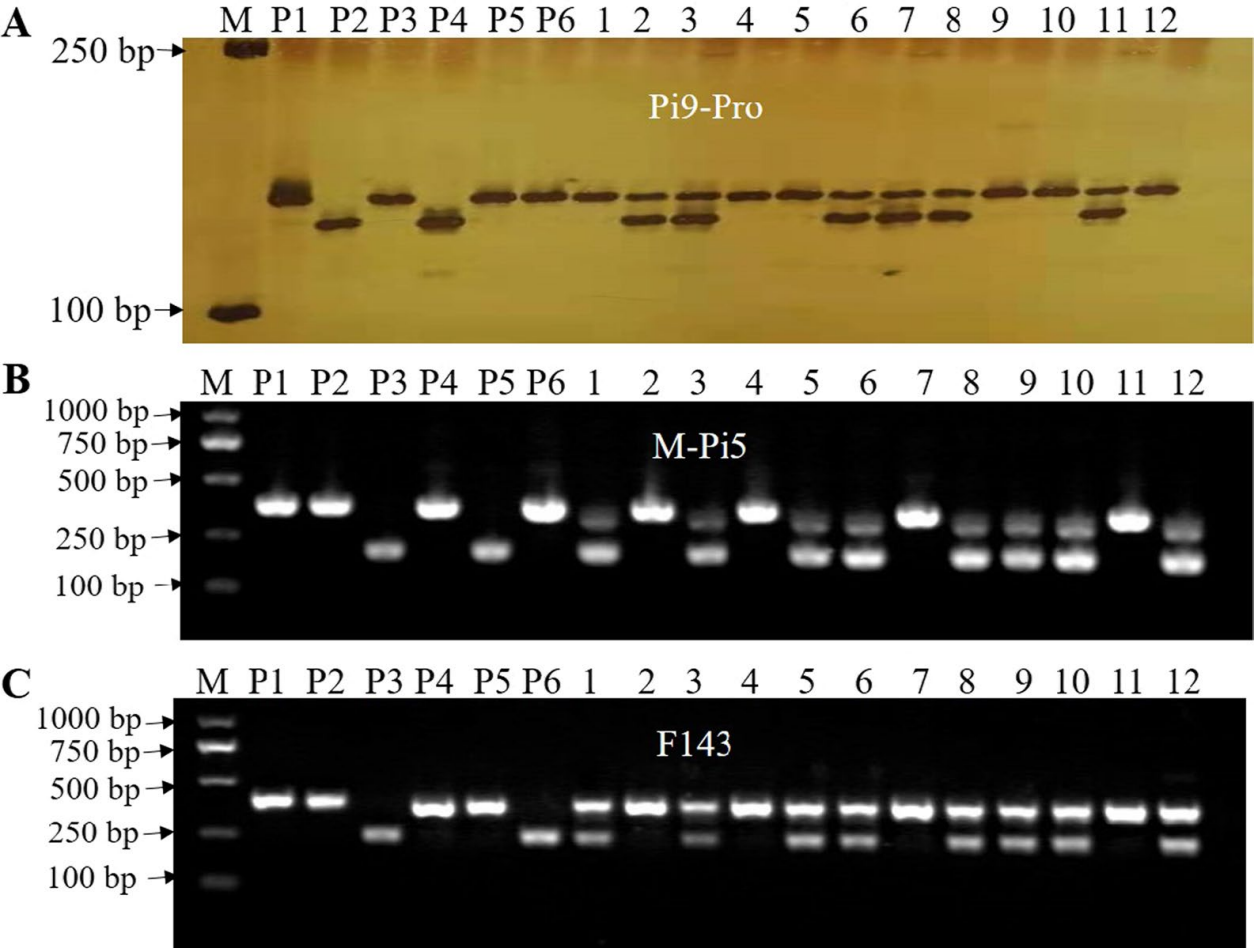
Selection of heterozygote plants with three genes in F_1_ population. **A** specific marker Pi9-Pro for *Pi9* were fractionated on 6% polyacrylamide gel; **B** specific marker M-Pi5 for *Pi5* were fractionated on 3% agarose gel; **C** specific marker F143 for *Pi54* were fractionated on 3% agarose gel; M: 2000 bp DNA Marker; P1: Huhan 1S; P2: Huhan 1B(Pi9); P3: Huhan 91(*Pi5* + *Pi54*); P4: 75-1-127 (*Pi9* donor parent); P5: IRBL5-M (*Pi5* donor parent); P6: IRBLkh-K3 (*Pi54* donor parent); lanes 1–12: 12 Plants of the F1 populations

Triple hybrids with heterozygous alleles of three blast resistance genes (*Pi9, Pi5,* and *Pi54*) were obtained from the crosses of Huhan 1S/Huhan 1B(*Pi9*)//Huhan 91 (*Pi5* + *Pi54*) (Fig. 1). Huhan 1B (*Pi9*), a breeding line carrying *Pi9* gene, and Huhan 91 containing *Pi5* and *Pi54* were the donors of rice blast resistance genes. Specific markers of the three resistance genes confirmed heterozygosity (Fig. 2, Additional file 1: Table S1). Positive F_1_ plants were self-pollinated to produce F_2_ plants. A total of 27 sterile plants carrying homozygous *Pi9*, *Pi5*, and *Pi54* genes were selected by phenotype and PCR analysis using gene-specific markers. The 27 selected plants were then ratooned, and the stubbles were placed under low-temperature conditions (about 21 °C for more than 12 days during the panicle initiation primordial stage). The selfed seeds were harvested from the ratooned stubbles. In the winter of 2014, the F_3_ generation was planted under natural drought conditions in Hainan, and 128 individual plants with good comprehensive agronomic traits and strong drought resistance were selected. The F_4_ generation was planted in Shanghai in the summer of 2015, then, combined with blast screening and agronomic trait inspection, 26 individual plants were selected. The selfed seeds of the selected plants were obtained by placing stubble cuts under low-temperature conditions, as mentioned earlier. Planting of the F_5_ generation was continued in Hainan during the winter of 2015. The F_6_ generation was planted in Shanghai in the summer of 2016, eight lines were identified as homozygous at the *Pi9, Pi5,* and *Pi54* loci. The F_7_ generation was planted in Hainan during the winter of 2016, and three lines with desirable field agronomic traits were selected. In the summer of 2017, lines with the best comprehensive agronomic traits and the strongest heterosis and numbered 17S2174 were selected in Shanghai. The line growth was continued, and seeds were propagated in Hainan during the winter of 2017, adhering to the technical appraisal in Shanghai in August 2018 and named Huhan 74S.

### Resistance Identification

The newly PTGMS line Huhan 74S, Huhan 1S, Huhan 1B(*Pi9*), Huhan 91(*Pi5* + *Pi54*), and LTH (susceptible check) were scored at the seedling stage by artificial inoculation with 14 blast isolates in the greenhouse. Leaf blast resistance of the test materials revealed that Huhan 74S was resistant to 12 blast isolates with a resistance frequency of 85.7%, a higher resistance frequency than donor parents (71.4% and 78.6%). However, the parent Huhan 1S was resistant to only five isolates with a resistance frequency of 35.7%. LTH (susceptible check) was susceptible to blast, with a susceptibility frequency of 100%. The results showed that the leaf blast resistance level of the newly developed TGMS line Huhan 74S was significantly improved (Table 1).

**Table 1:** Evaluation of newly developed PTGMS line Huhan74S, parents, and susceptible check for resistance to 14 blast isolates under artificial inoculation in the greenhouse

Blast isolates	Resistance evaluation				
	Huhan 74S	Huhan 1S	Huhan 1B (*Pi9*)	Huhan 91 (*Pi5* + *Pi54*)	LTH (susceptible check)
A1	MS	S	MS	S	S
A33	R	S	R	S	S
A49	R	MR	MS	MR	S
B1	MS	S	S	S	S
B13	R	S	R	R	S
B15	R	S	R	R	S
B5	R	MR	R	R	S
C13	R	R	R	R	S
D1	R	MS	R	R	S
D3	R	R	R	R	S
E1	R	MS	MS	R	S
E3	R	MS	R	R	S
F1	R	S	R	R	S
G1	R	R	R	R	S
Resistance frequency (%)	85.7	35.7	71.4	78.6	100.0

R, Resistant; MR, Medium Resistant; S, Susceptible; MS, Medium Susceptible

**Fig. 3:**
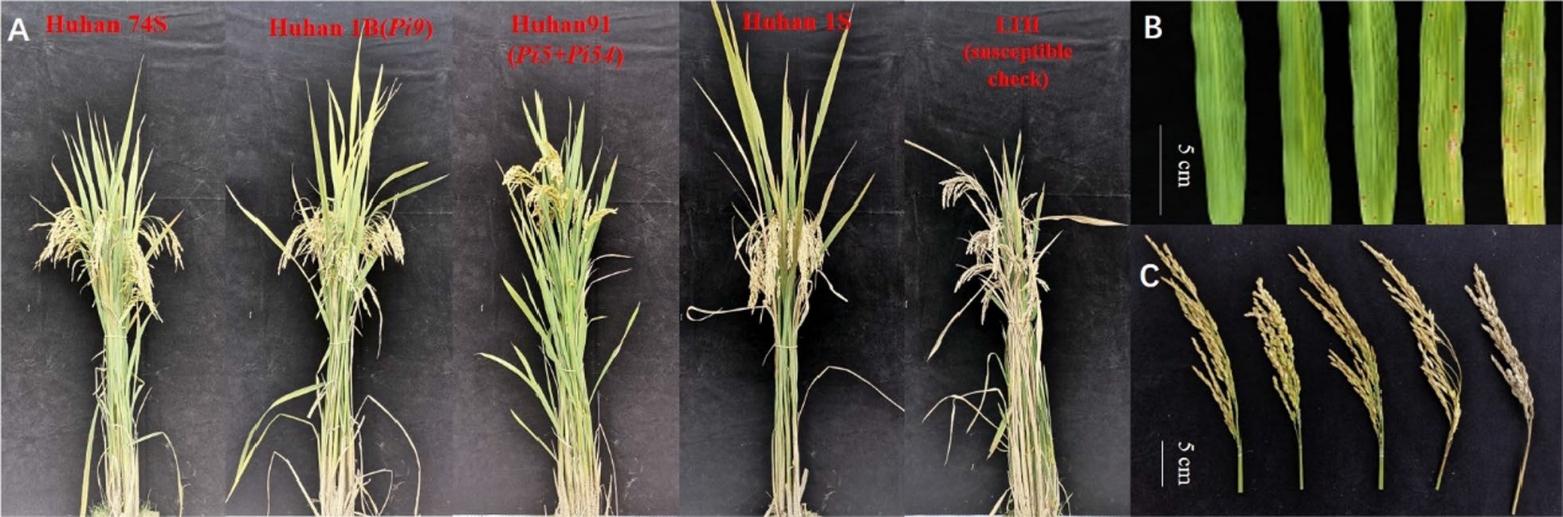
The performance for blast resistance of Huhan 74S, parents, and susceptible check in blast epidemic field. **A** The whole plant; **B** the leaf blast reactions; **C** the neck blast reactions; from the left to the right are Huhan 74S, Huhan 1B(*Pi9*), Huhan 91(*Pi5* + *Pi54*), Huhan 1S and LTH (susceptible check); Scale bars: 5 cm

Leaf and neck blast resistances are imperative for the practicability of breeding blast-resistance cultivars. To further determine the field resistance of the newly bred PTGMS line, we conducted natural induction identification at the blast epidemic area in Jinggangshan, Jiangxi Province. The blast epidemic area is a test site for evaluating blast resistance of the China national rice regional test varieties. The results revealed that Huhan 74S line was resistant to blast, with a score of 2 for leaf blast and 1 for neck blast. Donor parents Huhan 1B(*Pi9*) and Huhan 91(*Pi5* + *Pi54*) exhibited resistance to blast. However, Huhan 1S was susceptible to both leaf and neck blast (Table 2, Fig. 3).

**Table 2:** Evaluation of leaf and neck blast resistances of newly developed PTGMS line Huhan74S, parents, and susceptible check in blast epidemic fields

Lines	Score of leaf blast	Score of neck blast	Resistance evaluation
Huhan 74S	2	1	R
Huhan 1S	7	7	S
Huhan 1B (*Pi9*)	3	1	R
Huhan 91 (*Pi5* + *Pi54*)	3	1	R
LTH (susceptible check)	9	9	HS

R, Resistant; MR, Medium Resistant; S, Susceptible; HS, High Susceptible

Bidirectional selection is applied to obtain high yield potential and good drought resistance in WDR. To clarify the drought resistance of the newly bred PTGMS line, we evaluated the drought resistance of Huhan 74S according to the China National Agricultural Industry Standard (NY/T 2863–2015). The drought resistance index and score of Huhan 74S were 0.98 and 2, respectively, which was similar to the resistance level of Huhan 1S (Table 3, Additional file 2: Fig. S1).

**Table 3:** Evaluation of drought resistance of Huhan 74S and Huhan 1S

Lines	Drought resistance index	Score	Resistance evaluation
Huhan 74S	0.98	2	R
Huhan 1S	1.10	2	R
Hanyou 73 (CK)	1.00	2	R
IR36 (CK)	0.48	4	MS

R, Resistant; MS, Medium Susceptible

The above resistance identification outcomes indicated that the novel PTGMS line Huhan 74S, which aggregated three broad-spectrum blast resistance genes through MAS, was resistant to rice blast and retained the strong drought resistance.

### Fertility-Sterility Alteration Pattern in the Field and Agronomic Traits

**Fig.4:**
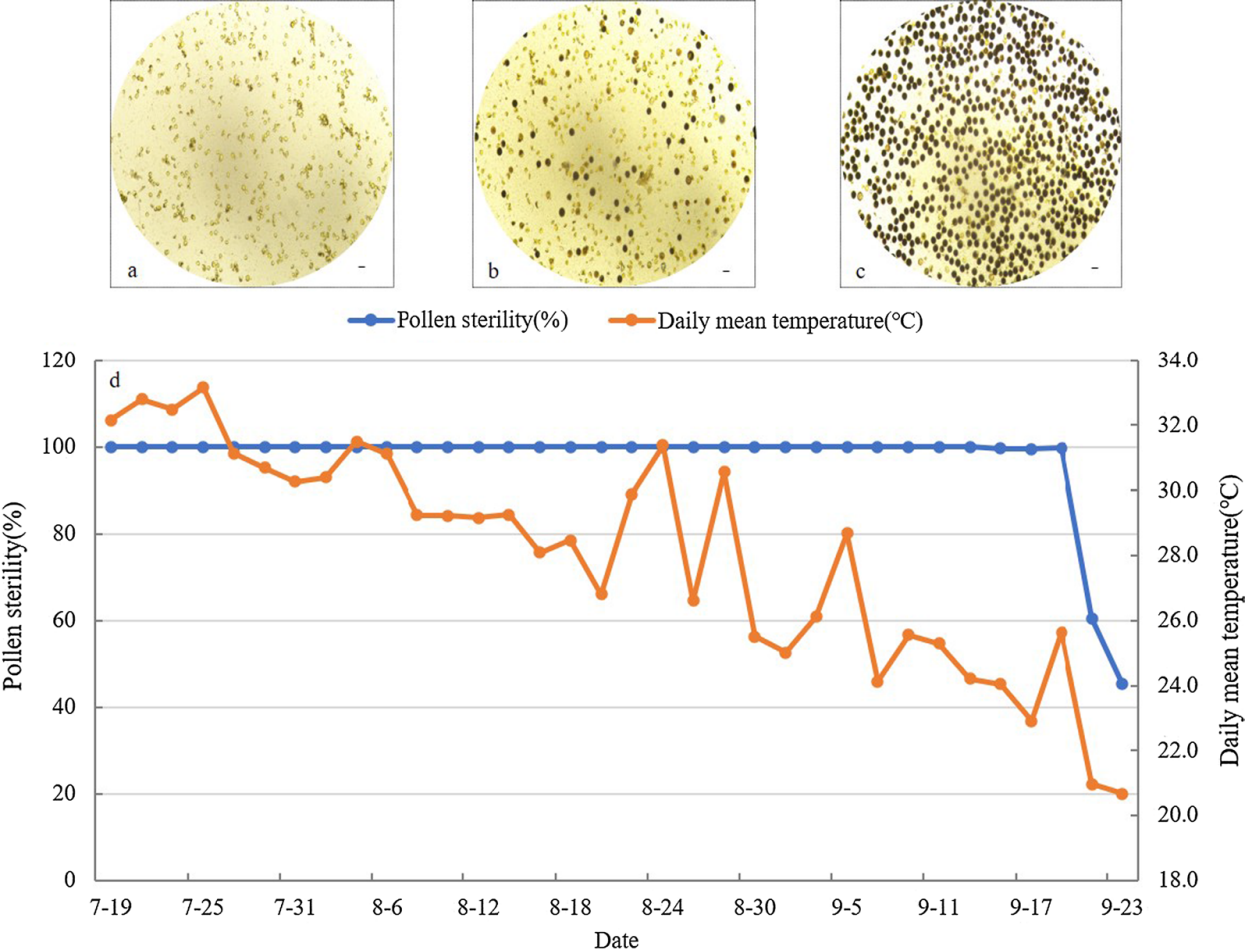
Fertility-sterility alteration pattern of Huhan 74S in 2017 at Shanghai.** a**–**c** Pollen grains from Huhan 74S on 12th August (**a**), 21st September (**b**) and 23th September (**c**). **d** Dynamic pollen sterility expressions of Huhan 74S relative to daily mean temperature (DMT) data between 19th July to 23th September in 2017 at Shanghai. Scale bars: 50 μm

Dynamic pollen grain fertility expression of Huhan 74S was observed in the summer of 2017 under natural field conditions. Between 19th July and 15th September, daily pollen microscopy outcomes showed no pollen, implying high abortion. Between 15 and 20th September, the pollen abortion type was typical abortion, with a small amount of round abortion. After 21st September, the pollen gradually normalized. These results indicate that Huhan 74S was completely male sterile (pollen sterility surpassing 99.5%) from 15th July to 20th September, possessing a stable sterile period of 67 days. Huhan 74S fertility alternated from completely sterile to partially fertile in late September when the temperature decreased, and daylength in early September became shorter (Fig. 4).

Huhan 74S has a compact plant type with strong stem and tillering ability, and medium spike (panicle) with a uniform grain setting. It was sown in Shanghai on May 15th, 2017, and headed on 5th August (82 days), exhibiting 81.3 cm plant height, 11.7 effective panicles per plant, 24.2 cm panicle length, 202.2 spikelets per panicle, 77.2% stigma exposure rate in sterile phase. In Hainan, Huhan 74S was sown on November 8th, 2017, and headed on February 29th, 2018 (101 days), exhibiting 86.2% seed setting rate and 1000 grain weight of 23.7 g. Rice grain quality analysis according to the NYT83-2017 standard showed that Huhan 74S met the quality requirements of third-class edible indica rice (Table 4).

**Table 4:** Agronomic performance of newly developed TGMS line Huhan 74S

Traits	Sterile phase in summer season of 2017 in Shanghai	Fertile phase in winter season of 2017–2018 in Hainan
Duration from sowing to heading (d)	82.0 ± 6.5	101.5 ± 2.5
Plant height (cm)	81.3 ± 4.3	80.1 ± 2.4
No. of panicles per plant	11.7 ± 1.0	15.2 ± 1.6
Panicle length (cm)	24.2 ± 1.3	23.3 ± 0.6
No. of Spikelets per panicle	202.2 ± 13.6	189.3 ± 9.4
Seed setting rate (%)	–	86.3 ± 3.7
1000-grain weight (g)	–	23.7 ± 0.6
Stigma exsertion percentage (%)	77.2 ± 12.5	–
Brown rice percentage (%)	–	79.3 ± 8.8
Milled rice percentage (%)	–	62.8 ± 6.2
Head rice percentage (%)	–	68.7 ± 5.5
Chalky rice percentage (%)	–	13.1 ± 1.1
Chalkiness degree	–	3.9 ± 0.2
Grain length (mm)	–	6.8 ± 0.4
Grain length/width ratio	–	3.3 ± 0.2
Alkali spreading value	–	6.0 ± 0.3
Amylose content (%)	–	14.3 ± 2.2
Gel consistency (mm)	–	65.4 ± 5.4

The values of agronomic traits are presented as means ± standard deviation

### Characteristics of Fertility-Sterility Alteration in the Artificial Climate Chamber

The critical temperature point (CTP) of fertility-sterility alteration is of great practical significance for safe seed production. Here, PTGMS plants were placed in five artificial climate chambers with daily mean temperatures (DMT) of 21 °C, 22 °C, 23 °C, 24 °C, and 25 °C from 5–16 days after the panicle initiation primordial stage. Light duration and relative humidity of the five growth chambers were uniformly maintained at 14 h and 75%, respectively. The pollen grains collected from the top five spikelets of each panicle per plant that headed during the 5–16 days after the end of the environmental treatment in the controlled climate chamber were examined under a microscope. Consequently, the pollen of the newly bred PTGMS line Huhan 74S was completely sterile (pollen sterility > 99.5%) in the growth chambers with 23 °C to 25 °C DMT, but partially fertile under 22 °C (Table 5). Therefore, two-line hybrid seed production should be conducted under conditions where DMT is higher than 23 °C.

**Table 5:** Fertility-sterility alteration behavior of Huhan74S and Huhan 1S under five different temperature regimes

Lines	Pollen sterility (%)				
	21 °C	22 °C	23 °C	24 °C	25 °C
Huhan 74S	60.5	98.6	100	100	100
Huhan 1S	58.5	98.2	100	100	100

### Agronomic Traits and Resistance Performance of Derived Hybrids

Five hybrid rice combinations were derived from the new PTGMS line Huhan 74S and its pollen parents, Huazhan, Chenghui 727, Hanhui No.3, Hanhui 808, Hanhui 8228. As a result, all combinations demonstrated satisfactory luxuriance and strong lodging resistance, with the yield potential reaching 9–10 t/hm^2^ (Table 6). This was equivalent to the control Fengliangyou No.4. The score of leaf blast in all combinations ranged from 1 to 3, the score of neck blast ranged from 1 to 3. Additionally, the drought resistance of the other four hybrid combinations was above medium resistance except that of Huhan74S/Chenghui727, which was drought-sensitive (Table 6).

**Table 6:** Agronomic characteristics and resistances of F1 hybrid derived from Huhan 74S

Combinations	Growth duration (d)	Plant height (cm)	Panicle length (cm)	No. of panicles per plant	No. of grains per panicle	Seed setting rate (%)	1000-grain weight (g)	Grain yield (t/ha)	Blast resistance			Drought resistance	
									Score of leaf blast	Score of neck blast	Evaluation	Score	Evaluation
Huhan 74S/Huazhan	114.0 ± 1.5	107.7 ± 2.4	23.2 ± 2.2	14.2 ± 1.1	186.5 ± 11.5	83.0 ± 5.5	22.9 ± 0.7	9.6 ± 1.3	1	1	R	3	MR
Huhan 74S/Chenghui 727	118.5 ± 2.0	112.3 ± 1.4	26.1 ± 1.7	14.5 ± 1.4	169.5 ± 9.7	87.8 ± 6.3	24.2 ± 0.4	9.3 ± 0.5	2	3	MR	4	MS
Huhan 74S/Hanhui 3	115.0 ± 2.0	116.7 ± 3.2	24.3 ± 0.8	11.4 ± 0.5	132.2 ± 9.9	88.8 ± 5.9	25.3 ± 1.1	9.3 ± 0.9	3	3	MR	2	R
Huhan 74S/Hanhui 808	118.5 ± 1.0	108.0 ± 1.6	24.5 ± 1.6	11.7 ± 2.1	187.0 ± 14.8	87.2 ± 4.6	25.6 ± 0.9	9.7 ± 1.5	2	1	R	2	R
Huhan 74S/Hanhui 8228	123.5 ± 1.5	110.3 ± 3.1	22.9 ± 2.5	13.6 ± 2.3	121.2 ± 8.4	91.3 ± 4.4	25.1 ± 1.0	10.3 ± 1.7	3	3	MR	2	R
Fengliangyou 4(CK)	128.0 ± 1.5	124.3 ± 3.8	24.7 ± 0.9	13.1 ± 1.0	154.2 ± 10.2	90.0 ± 5.2	27.2 ± 0.8	9.5 ± 0.7	8	7	S	5	S

R, Resistant; MR, Moderate resistant; S, Susceptible; MS, Moderate Susceptible. The values of agronomic traits are presented as mean ± standard deviation

## Discussion

Hybrid rice has been successfully used for commercial rice production for 40 years in China (Xie and Zhang 2018). Compared with three-line hybrid rice, the two-line hybrid rice breeding system based on PTGMS line has some advantages: (1) Male sterility is controlled by recessive nuclear genes, any genotype with good combining ability can be used as the male parent, thus a wide selection range of restorer line. Also, the probability of obtaining heterosis by test crossing is higher than that by three-line method; (2) The PTGMS line remains unaffected by the relationship between restoration and maintenance, this is specifically suitable for breeding intersubspecific hybrid (*indica/japonica*); (3) The cost of hybrid seed production is lower than that of three-line seed production, and the procedure of hybrid seed production is simplified by dual-use of one line. As an effective method of enhancing rice yield and quality, two-line hybrid breeding is widely used in China (Yang et al. 2007; Yuan 2017). Nevertheless, the productivity of the two-line hybrid rice is severely limited by diseases, among which blast is considered the most destructive, causing huge yield losses (Ashkani et al. 2016). Molecular marker-assisted selection (MAS) is an important approach for disease resistance breeding. Particularly, the use of cloned genes significantly reduces the breeding period and cost (Jiang et al. 2012; Ishihara et al. 2014; Kumar et al. 2016). At present, the improvement of blast resistance of two-line male sterile lines is primarily based on marker-assisted selection of a single or two genes. Jiang et al (2015a) introduced the broad-spectrum and long-lasting rice blast resistance gene (*Pi2*) into the excellent thermosensitive genetic male sterile line (TGMS) C815S and selected four TGMS lines with blast resistance using dual selection approach of combining phenotypic and genotype selection with background selection. Based on Guangzhan 63-4S, Jiang et al (2015b) further bred a two-line sterile line with two resistance genes (*Pi2* and *Xa23*) using molecular markers. Yang et al (2019) effectively introduced blast resistance gene *Pi2* into PTGMS line Feng39S by MAS, resulting in improved male sterile line and hybrid combination significantly enhanced the blast resistance. In the practice of crop disease resistance breeding, repeated use of a single gene for a long time leads to the loss of resistance. Multigene pyramiding is conducive to broadening the resistance spectrum and enhancing the resistance of crops. Resistance gene pyramiding breeding prevents the production risk caused by loss of resistance in varieties with a single resistance gene. Huhan 1S is a WDR PTGMS line with strong drought resistance; however, its application has been limited due to its poor blast resistance. This study introduced three blast resistance genes *Pi9*, *Pi5*, and *Pi54,* into Huhan 1S through MAS and phenotypic selection, and a new PTGMS line Huhan 74S was developed. The polymerized blast resistance genes were broad-spectrum resistance genes at different loci, conducive for enhancing a stable and durable resistance of rice varieties. The results of artificial inoculation at the seedling stage under natural blast epidemic field conditions showed a significant increase in the blast resistance of Huhan 74S and its combinations.

Rice production is usually affected by biotic and abiotic stresses. Zhang (2007) introduced the concept of Green Super Rice (GSR), a development direction of rice in the future, aimed towards integrating all green traits into rice varieties to cope with the challenges of resources and the environment. Less pesticide, less chemical fertilizer, water-saving and drought resistance, acceptable grain quality, and high yield should be met during the production of GSR. Notably, WDR is a vital component of the GSR plan, and its development changes the conventional rice planting mode to achieve resource-saving and environment-friendly (Luo 2010). Drought resistance is a complex trait affected by thousands of drought resistance genes and their interaction with the environment. The formation of drought resistance is caused by the interaction of multiple drought resistance genes rather than a single drought resistance gene (Shinozaki and Yamaguchi 2007; Hadiarto and Tran 2011). Xia et al (2019) proposed a model for adaptive differentiation between the upland and lowland rice during domestication. This model, particularly in the evolution of drought resistance in the upland rice and its balance with productivity, has been proven to be partially true in our efforts to develop WDR cultivars, where the bidirectional selection is applied to obtain both high yield potential and good drought resistance. Wei et al. reported that under high-intensity stress selection in the target environment, several genes of conventional hybrid combinations in drought adaptation and drought resistance transcriptional regulatory network were retained and pyramided in the genome of breeding offspring. This significantly enhanced the drought resistance of Huhan 2B (Wei et al. 2016, 2017). In the present study, we did not use the backcross breeding approach. In addition to the use of molecular markers to ensure the prospect selection of resistance genes, alternative screening of drought resistance, disease resistance, yield, and quality traits conducted in the segregated generation population. This was geared towards significantly retaining the characteristics of water-saving and drought resistance, as well as high yield and quality traits. The agronomic traits and drought resistance of the newly bred PTGMS line Huhan 74S were similar to those of Huhan 1S. Moreover, the blast resistance of Huhan 74S was significantly enhanced, thereby verifying the high efficacy of molecular markers combined with stress selection.

The critical temperature point (CTP) of fertility-sterility alteration of PTGMS line is the change of pollen from fertile to sterile or vice versa at a certain temperature. Whereas a stable sterility duration (SSD) is the time (d) when pollen remains completely sterile at a specific place (Virmani et al. 2003). These two parameters are significant for safe seed production of two-line hybrid rice. Here, the CTP of Huhan 74S was at 23 °C of DMT, and the SSD was 67 days. Therefore, Huhan 74S was used for seed production when the DMT was greater than 23 °C at the booting stage and to breed PTGMS line below 23 °C (Fig. 2, Table 5). The rice grain quality indexes of Huhan 74S met the quality requirements of third-class, and the yield potential of the combinations was high. Therefore, Huhan 74S passed the technical identification of Shanghai in August 2018 and has been incorporated into the programs of other breeding institutes in China, indicating a broad application prospect.

## Conclusion

This study employed molecular marker-assisted selection (MAS), conventional hybridization, and high-intensity stress screening to integrate the broad-spectrum blast resistance genes *Pi9*, *Pi5*, and *Pi54* into Huhan 1S, an elite water-saving and drought-resistance rice PTGMS line. This led to the development of Huhan 74S, which together with its derived hybrids showed resistance to rice blast and drought. Notably, Huhan 74S had a critical temperature point of fertility-sterility alteration and high rice grain quality. Thus, the newly developed PTGMS line Huhan 74S with *Pi9*, *Pi5*, and *Pi54* genes is currently being utilized for heterosis breeding of broad-spectrum blast resistant two-line WDR hybrids. It can also serve as an improved disease donor source for further PTGMS parental line improvement.

## Materials and Methods

### Plant Materials

Huhan 1S, a WDR two-line male sterile line, Y58S was used as PTGMS gene donor. Huhan 1B(*Pi9*), was a near-isogenic line of the *Pi9* gene in Huhan 1B genetic background (using Huhan 1B as the recurrent female parent and 75-1-127 as the donor line). Huhan 91(*Pi5* + *Pi54*), a blast resistance breeding line, was from ‘Huhan 3/IRBL5-M//Xiushui 123/IRBLkh-K3’ in our laboratory. IRBL5-M and IRBLkh-K3 were used as the donor parents for blast resistance genes *Pi5* and *Pi54*, respectively. Restorer lines (Huazhan, Chenghui 727, Hanhui No.3, Hanhui 808, and Hanhui 8228) were used to identify the heterosis. The blast susceptible check was Lijiang Xintuan Heigu (LTH), drought resistance check was Hanyou 73, drought-sensitive check was IR36, whereas the heterosis identification control was Fengliangyou No.4. All plant materials were provided by Shanghai Agrobiological Gene Center (SAGC).

### DNA Extraction, Gene-Specific Markers, and PCR Amplification

Genomic DNA was extracted from fresh young leaves at the peak tillering stage using the Cetyltrimethylammonium Bromide (CTAB) method (Murray and Thompson 1980). The details of gene-specific markers are provided in Additional file 1. The primers used were synthesized by Sangon Biotech (Shanghai) Co., Ltd. The PCR was performed in a 20-µl reaction volume, containing 2 μl of genomic DNA (10 ng/μl), 10 uL of Taq PCR master mix (Tiangen BioTech (Beijing) Co., Ltd.), 0.5 uL of each primer (10 uM), and 7 µl of dd H_2_O. The PCR procedure involved: pre-denaturation at 95 °C for 5 min, then 30 cycles at 95 °C for 30 s, 55 °C for 30 s, and 72 °C for 1 min, and final extension at 72 °C for 5 min. The amplified products were electrophoresed in a 3% agarose gel stained with ethidium bromide or in a 6% polyacrylamide gel in 0.5 × TBE buffer (Creste et al. 2001).

### Evaluation of Rice Blast Resistance

To detect the resistance spectrum of rice blast, we artificially inoculated the test plants with 14 prevalent isolates of *M. oryzae* collected from Zhejiang, Hubei, Hunan, Jiangxi and Anhui provinces of China (ZA1, ZA33, ZA49, ZB1, ZB5, ZB13, ZB15, ZC13, ZD1, ZD3, ZE1, ZE3, ZF1, and ZG1) at the seedling stage under greenhouse conditions. The isolates were cultured in oat medium (oatmeal 30 g/L, tomato juice 150 mL/L, agar powder 20 g/L, pH 6.5) for 3–5 days at 28 °C in the dark. Thereafter, they were cultured under constant light at 26 °C for 5–6 days to form conidia. The mature conidia were washed with sterile water to prepare an inoculum with a concentration of about 1 × 10^5^ cfu/ml. The seedlings were inoculated with the prepared spore solution by artificial spray during the three-leaf stage. The inoculated isolates were placed in a dark room at 25 °C and 90% humidity for 24 h, then transferred into the growth chamber to grow for six days at 25–28 °C and 90% humidity under 12/12 h (bright/ dark) photoperiod. The disease was investigated after 7 days of inoculation. The natural disease nursery of rice blast was selected at Jinggangshan identification nursery in Jiangxi Province. Disease reaction was evaluated based on the 0–9 grading standard of the International Rice Research Institute (IRRI) (IRRI 2002).

### Evaluation of Drought Resistance

The experiment was conducted in the winter of 2017 at the experimental field of the Shanghai Agrobiological Gene Center in Lingshui County, Hainan Province. Two treatments, including drought and control, were set for drought resistance evaluation. The test materials were replicated three times; nine rows were planted in each plot, with nine plants in each row. The row spacing was 20 cm × 23 cm. Intermittent irrigation was used to keep the field moist from seeding to stage II of panicle differentiation, without leaving any water layer. The panicles were subjected to drought stress at stage II of differentiation. The stress was halted for drought-sensitive rice check variety when all leaf curling failed to recover for more than five days or when the leaf death rate reached 50% in the morning. Thereafter, the field water management was restored. The control treatment was set up in the adjacent paddy field, and the whole growth period was as per conventional paddy field cultivation and management. The yield of plots was measured at the maturity stage. The drought resistance index was calculated according to Technical Specification of Identification and Evaluation for Rice Drought Resistance (NY/T 2863–2015) (http://hbba.sacinfo.org.cn/). Drought resistance levels were classified based on drought resistance index as High Resistant (≥ 1.30), Resistant (0.90–1.30), Medium Resistant (0.70–0.89), Medium Susceptible (0.35–0.69), and Susceptible (≤ 0.35).

### Field Fertility Survey

The planting was executed in stages during the summer of 2017 at the Zhuanghang Experimental Station of the Shanghai Academy of Agricultural Sciences. From April 20th, the planting was conducted at ten-day intervals, where seven stages were planted. Single seedlings were planted in three rows, with a spacing of 20 cm × 16.7 cm between rows. In each row, 12 plants were planted. This experiment applied conventional field water and fertilizer management. From the earliest panicle of the test materials, samples were collected daily for pollen microscopic examination to observe the pollen fertility and record the pollen sterility data and temperature. Five panicles were collected from each line. Analysis of pollen sterility data in relation to temperature weather charts was performed to determine the CTP of fertility-sterility alteration under natural conditions.

### Agronomic and Rice Grain Quality Traits

The agronomic traits of the newly bred PTGMS line were investigated during the summer and winter of 2017 at Shanghai and Hainan, respectively. We examined the duration (from sowing to heading), plant height, number of panicles per plant, panicle length, number of spikelets per panicle, and stigma exsertion percentage during the sterile phase at Shanghai. Meanwhile, the seed setting rate and 1000-grain weight were investigated during the fertile phase at Hainan. The rice grain quality traits were evaluated after storing the harvested and dried rice at room temperature for three months. The evaluated quality parameters included brown rice percentage, milled rice percentage, head rice percentage, chalky rice percentage, chalkiness degree (%), grain length (Mm), grain length/width ratio, alkali spreading value, amylose content (%), and gel consistency (Mm). The detection of various indicators was executed based on the Determination of Rice Quality (NYT83-2017) (http://hbba.sacinfo.org.cn/).

### Characterization of the Newly Bred PTGMS Line for Fertility-Sterility Alternation in Growth Chambers

In the summer of 2018, the newly bred PTGMS line was sown at the experimental field of Huazhong Agricultural University. Uniform and healthy rice seedlings at the five-leaf stage (about 25 days after sowing) were selected for transplantation. Five plants were planted in each pot, and plastic labels were attached to each plant. Five plant growth chambers (model: ZSX1500GS, Shanghai Jing Wins and Scientific Equipment Co., Ltd., China) were adjusted for trial operation a week before the actual use in the experiment. All the five plant growth chambers had 14 h light, relative humidity of 75%, and average daily temperatures of 21 °C, 22 °C, 23 °C, 24 °C, and 25 °C. The plants were placed in growth chambers from 5 to 16 days after the panicle initiation primordial stage. The plants were removed from the growth chambers after 12 days of temperature treatment. The pollen grains of the first five spikelets of each plant that headed 5–16 days after the end of the treatment were observed under a microscope. Based on pollen morphological classification, the pollen grains were stained with I_2_-KI, and the degree of pollen sterility per panicle was recorded (Virmani et al. 2003). Lines with an average pollen sterility rate above 99.5% were completely sterile.

### Evaluation of Derived Hybrids

The novel PTGMS line was used as a female parent to produce F_1_seeds with five male parents, including Huazhan, Chenghui 727, Hanhui No.3, Hanhui 808, and Hanhui 8228. Fengliangyou No.4 was used as the control hybrid. Hybrids were transplanted in the summer of 2018 at the Zhuanghang Experimental Station of Shanghai Academy of Agricultural Sciences. The comparison experiment was arranged in random groups and repeated three times. Each plot was planted in five rows, with 12 plants in each row and the row spacing of 20.0 cm × 16.7 cm. The entire growth period was recorded. After maturation, the middle five plants were collected to examine the plant height, panicle length, number of panicles per plant, number of grains per panicle, seed setting rate, and 1000-grain weight. The plots were manually harvested and calculated as actual yield.

## Supplementary Information

**Additional file 1: Table S1**. Gene specific markers used in this study.

**Additional file 2: Fig S1**. Drought resistance levels of Huhan 74S, Huhan 1S, and control varieties in identification facility greenhouse.

## Data Availability

All relevant data are provided in tables within the paper in the additional files.

## Abbreviations

PTGMS: Photoperiod and thermo-sensitive genic male sterile
CMS: Cytoplasmic male sterility
MAS: Marker-assisted selection
WDR: Water-saving and drought resistance rice
GSR: Green super rice
CTP: Critical temperature point
DMT: Daily mean temperature
