# Supplementary material for: Molecular Breeding of a Novel PTGMS Line of WDR for Broad-Spectrum Resistance to Blast Using Pi9, Pi5, and Pi54 Genes
Source: Rice (N Y). 2021 Nov 25;14:96. doi: 10.1186/s12284-021-00537-1 (PMC8617131; doi:10.1186/s12284-021-00537-1)
Supplement: Supplementary file 1 — Additional file 1: Table S1. Gene specific markers used in this study. [file 12284_2021_537_MOESM1_ESM.docx]

| Table S1 Gene specific markers used in this study | | | | | | | | |
| --- | --- | --- | --- | --- | --- | --- | --- | --- |
| Gene | Chr | Position(bp) | Marker | Forward (5'–3') | Reverse (5'–3') | Polymorphic fragment size | Mark type | Reference |
| *Pi9* | 6 | 10381428-10381565 | Pi9-Pro | TGATTATGTTTTTTATGTGGGG | ATTAGTGAGATCCATTGTTCC | 128bp(R)/138bp(S) | gene specific marker | Tian et al, 2016 |
| *Pi5* | 9 | 9668284-9668590 | M-Pi5 | ATAGATCATGCGCCCTCTTG | TCATACCCCATTCGGTCATT | 206bp(R)/307bp(S) | gene specific marker | Gao et al, 2010 |
| *Pi54* | 11 | 25264124-25264524 | F143 | CCCAACATTGGTAGTAGTGC | TCCTTCATACGCAACAATCT | 258bp(R)/401bp(S) | gene specific marker | Wang et al, 2014 |
| Note: R: Resistant; S: Susceptible | | | | |  |  |  |  |
