# Supplementary material for: Molecular Breeding of a Novel PTGMS Line of WDR for Broad-Spectrum Resistance to Blast Using Pi9, Pi5, and Pi54 Genes
Source: Rice (N Y). 2021 Nov 25;14:96. doi: 10.1186/s12284-021-00537-1 (PMC8617131; doi:10.1186/s12284-021-00537-1)
Supplement: Supplementary file 2 — Additional file 2: Fig S1. Drought resistance levels of Huhan 74S, Huhan 1S, and control varieties in identification facility greenhouse. [file 12284_2021_537_MOESM2_ESM.docx]

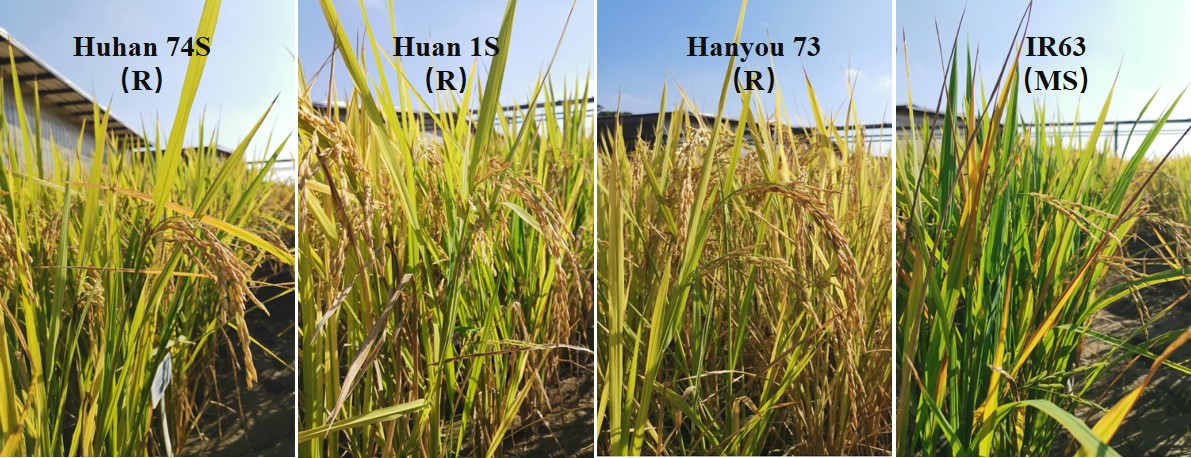


Fig. S1 Drought resistance levels of Huhan 74S, Huhan 1S, and control varieties in identification facility greenhouse. The panicles were treated with drought stress at stage II of differentiation. For drought-sensitive rice check varieties, the stress was halted when all leaf curling failed to recover for more than five days or the leaf dead rate reached 50% in the morning. Thereafter, the field water management was restored. R: Resistant; MS: Medium Susceptible.
